# Global Gene-Expression Analysis to Identify Differentially Expressed Genes Critical for the Heat Stress Response in *Brassica rapa*

**DOI:** 10.1371/journal.pone.0130451

**Published:** 2015-06-23

**Authors:** Xiangshu Dong, Hankuil Yi, Jeongyeo Lee, Ill-Sup Nou, Ching-Tack Han, Yoonkang Hur

**Affiliations:** 1 Department of Biology, College of Biological Science and Biotechnology, Chungnam National University, Daejeon, Republic of Korea; 2 Department of Horticulture, Sunchon National University, Suncheon, Jeonnam, Republic of Korea; 3 Department of Life Science, Sogang University, Seoul, Republic of Korea; Ajou University, REPUBLIC OF KOREA

## Abstract

Genome-wide dissection of the heat stress response (HSR) is necessary to overcome problems in crop production caused by global warming. To identify HSR genes, we profiled gene expression in two Chinese cabbage inbred lines with different thermotolerances, Chiifu and Kenshin. Many genes exhibited >2-fold changes in expression upon exposure to 0.5– 4 h at 45°C (high temperature, HT): 5.2% (2,142 genes) in Chiifu and 3.7% (1,535 genes) in Kenshin. The most enriched GO (Gene Ontology) items included ‘response to heat’, ‘response to reactive oxygen species (ROS)’, ‘response to temperature stimulus’, ‘response to abiotic stimulus’, and ‘MAPKKK cascade’. In both lines, the genes most highly induced by HT encoded small heat shock proteins (Hsps) and heat shock factor (Hsf)-like proteins such as *HsfB2A* (Bra029292), whereas high-molecular weight Hsps were constitutively expressed. Other upstream HSR components were also up-regulated: ROS-scavenging genes like *glutathione peroxidase 2* (*BrGPX2*, *Bra022853*), protein kinases, and phosphatases. Among heat stress (HS) marker genes in *Arabidopsis*, only *exportin 1A* (*XPO1A*) (*Bra008580*, *Bra006382*) can be applied to *B*. *rapa *for basal thermotolerance (BT) and short-term acquired thermotolerance (SAT) gene. *CYP707A3* (*Bra025083*, *Bra021965*), which is involved in the dehydration response in *Arabidopsis*, was associated with membrane leakage in both lines following HS. Although many transcription factors (TF) genes, including *DREB2A* (*Bra005852*), were involved in HS tolerance in both lines, *Bra024224 *(*MYB41*) and *Bra021735 *(a bZIP/*AIR1 *[*Anthocyanin-Impaired-Response-1*]) were specific to Kenshin. Several candidate TFs involved in thermotolerance were confirmed as HSR genes by real-time PCR, and these assignments were further supported by promoter analysis. Although some of our findings are similar to those obtained using other plant species, clear differences in *Brassica rapa* reveal a distinct HSR in this species. Our data could also provide a springboard for developing molecular markers of HS and for engineering HS tolerant *B*. *rapa*.

## Introduction

Heat stress (HS) and high temperature (HT) disturb cellular homeostasis and can interfere with various plant metabolic and physiological processes, including photosynthesis, growth, development, pollen fertility, and productivity in both crop species and model plants [1–3]. Therefore, global warming will threaten food safety by reducing crop production [4]. Understanding the molecular mechanisms underlying the heat stress response (HSR) will be necessary for the development of heat-tolerant crop plants capable of sustainable productivity in a changing environment.

Plants can perceive HS in various ways, and they tolerate or acclimate to heat *via* activation of signaling pathways, expression of stress proteins, and production of defensive materials. In particular, the activation of HSR genes, such as heat stress transcription factors (Hsfs), heat shock proteins (Hsps), and some other transcription factors (TFs), are very important for heat tolerance [5–7]. The activation of HSR genes begins with the perception of HS by a plasma membrane channel and various secondary messengers, such as calcium ions (Ca^2+^), nitric oxide (NO), and hydrogen peroxide (H_2_O_2_). In addition, a histone sensor in the nucleus and unfolded protein sensors present in the ER (endoplasmic reticulum) and cytosol play important roles in the perception process. These signals are then transmitted to downstream components, such as calmodulins, mitogen-activated protein kinases, Hsp90, and Hsfs. Finally the signals induce expression of HSR transcription factors (TFs) like WRKY, Dehydration-Responsive-Element Binding Factor (DREB), and bZIP [3,6–9]. Chaperones for protein homeostasis, osmolytes, and secondary metabolites responsible for ROS detoxification and osmoprotection are also important for thermotolerance [9].

Many Hsfs and Hsps play key roles in plant heat tolerance. The rapid accumulation of Hsps, which is required to protect the cell against HS conditions, is primarily controlled by Hsfs [10,11]. HsfA1s are major regulators in the HSR of tomato and *Arabidopsis* [12,13], HSfA2 is another major HS regulator in plants [10,14], and HsfA4 acts as a sensor of the H2O2 signal [15], whereas HsfA5 is a negative component of this pathway [16]. Hsps have been implicated in numerous cellular processes including protein folding, assembly, translocation, and degradation [17]. Hsps are classified according to their molecular weights into six major families: Hsp100 (Clp), Hsp90, Hsp70 (DnaK), Hsp60 (chaperonin and GroEL), Hsp40 (DnaJ), and Hsp20 (small HSP; sHsp). Chaperone functions prevent protein aggregation and misfolding, and are mainly mediated by Hsp70s, Hsp90s, and Hsp60s, whereas Hsp20s and Hsp100s are involved, respectively, in protection against protein aggregation and resolubilization of protein aggregates [18,19].

Identification of HSR genes from suitable genotypes can provide a springboard to the development of HS tolerance mechanisms. Omics approaches (genomics, transcriptomics, epigenomics, proteomics, and metabolomics) have been widely applied to investigations of plant HSR, and these analyses have identified many HSR genes [9]. Genome-wide identification of genes required for acquired thermotolerance have shown that expression of genes involved in protection of proteins, translation, and limiting oxidative stress is elevated, whereas expression of genes involved in programed cell death, basic metabolism, and biotic stress responses is reduced [20].

Chinese cabbage (*Brassica rapa* ssp. *pekinensis*) is one of the most important leafy vegetable crops in Korea and other eastern Asian countries, including China. Two inbred lines of Chinese cabbage, Chiifu and Kenshin, originated in different geographic regions: Chiifu originated in temperate regions, and Kenshin in subtropical and tropical regions [21]. Therefore, Kenshin has been traditionally used as breeding stock to develop heat-tolerant plants [22,23]. So far, most studies of HS have focused on cereals and model plants. Transcriptomics studies of stress responses have been performed in *B*. *rapa* in recent years, but these have primarily focused on cold, salt, and drought stresses [24–30]. Previously, the expression pattern of HSR genes in Chinese cabbage had been analyzed using 24K microarray, which does not include enough genes to cover the entire *B*. *rapa* genome [31]. In this study, we used version 3 microarrays (Br135K) to analyze gene expression in the Chiifu and Kenshin lines. We identified differentially expressed genes (DEGs), specifically expressed genes (SEGs), HSR genes, HS marker genes, and membrane leakage-related (MLR) genes, and we discuss these genes in the context of HS.

## Materials and Methods

### Plant materials and heat treatment

Seeds of two Chinese cabbage (*Brassica rapa* ssp. *pekinensis*) inbred lines, Chiifu and Kenshin, were obtained from the Korea *Brassica rapa* Genome Resource Bank, and the plants were grown for 4 weeks in a growth chamber at 22°C under a 16 h light/8 h dark photoperiod with a photon flux density of 140 μmol m^-2^ s^-1^. For heat shock treatments, leaf discs (1 cm in diameter) were incubated at 45°C for 0.5, 1, 2, 3, or 4 h by floating on a water bath. For control samples, the leaf discs were incubated at 22°C for 0.5h by floating on distilled water. Then, leaf discs were blotted, frozen immediately in liquid nitrogen, and stored at -70°C.

### Electrolyte leakage test

Immediately after treatment, electrolyte leakage from HS-treated and control plants was measured as previously described [32,33] with some modifications [28]. Briefly, five leaf discs were randomly selected from mixed discs after excision from fully expanded leaves of six plants (10 discs/plant and mixed), and placed in a glass tube with 10 ml distilled water. The samples were then incubated on an orbital shaker at 150 rpm for 30 min at room temperature, after which the initial conductivity (I) was measured using a CON110 conductivity meter (Oakton Ins. USA). The leaf discs were then kept in a boiling water bath for 10 min, after which they were cooled to room temperature, and the final conductivity (F) was measured. The relative electrolyte leakage was calculated using the formula I/F ×100.

### Construction of Br135K chips

The Br135K microarray (Brapa_V3_microarray, 3′-Tiling microarray) is a high-density DNA array prepared by NimbleGen (http://www.nimblegen.com/) using Maskless Array Synthesizer (MAS) technology as described in Jung et al. [28]. Labeling, data processing, and background correction were performed as described previously [28]. To assess the reproducibility of the microarray analysis, we repeated the experiment using independently prepared total RNA from two or three biological replicates. The complete raw microarray data have been deposited in the Omics database of NABIC (http://nabic.rda.go.kr) with accession numbers NC-0023-000001–NC-0023-000024.

### Gene chip data analysis

Genes with adjusted P-value or false discovery rate (FDR) below 0.05 were collected and further selected for those genes with expression greater than or less than in at least one treatment. Multivariate statistical tests such as principal component analysis and multidimensional scaling were performed with Acuity 3.1 (Molecular Devices, USA). Clustering analysis was carried out using MultiExperiment Viewer version 4.9 (MeV4.9, http://www.tm4.org/mev.html). To obtain insights regarding the putative biological functions and biochemical pathways of DEGs, we carried out enrichment analyses by searching Gene Ontology (GO) [34], agriGO [35], and the Kyoto Encyclopedia of Genes and Genomes [36].

### RNA extraction, RT-PCR, and qRT-PCR analysis

Total RNA was treated with RNase-free DNase I (Promega, USA) to remove genomic DNA contamination. First-strand cDNA synthesis was carried out using the Ace-α kit (Toyobo, Osaka, Japan). RT-PCR was performed using 25 ng of cDNA from plants exposed to HS treatments. The gene-specific primers for the stress-responsive genes are listed in S1 Table. Reactions were performed in 0.2 mL PCR tubes containing 10 pmol of each primer, 150 μM of each dNTP, 1.2 U of *Taq* polymerase, and 1X *Taq* polymerase buffer; double-distilled water was added to a total volume of 20 μL. The PCR cycle consisted of pre-denaturation at 94°C for 5 min, followed by 25 cycles of denaturation at 94°C for 30 s, annealing at 58°C for 30 s, and extension at 72°C for 45 s, followed by an additional extension step for 5 min at 72°C. PCR products were electrophoresed on 1.5% agarose gels. For qRT-PCR, pre-denaturation was performed at 95°C for 30 seconds, followed by a 40-cycle three-step reaction (5 seconds at 95°C, 20 seconds at 60°C, and 15 seconds at 72°C) with the primer sets shown in S2 Table.

### Analysis of enriched conserved cis-elements

HSR genes were subjected to the Short Time-series Expression Miner (STEM) [37], and 1,000 bp upstream sequences of DEG start codons were analyzed for the presence of plant *cis*-acting elements (PLACE) [38]. The occurrences of these motifs were compared with their frequencies among all promoters represented in the 135K microarray. A P-value was then calculated for each motif and profile combination, based on the hypergeometric distribution [39]. Motifs with P-values below 10^-4^ were considered to be significantly enriched.

## Results and Discussion

### Membrane leakage test

**Fig 1:**
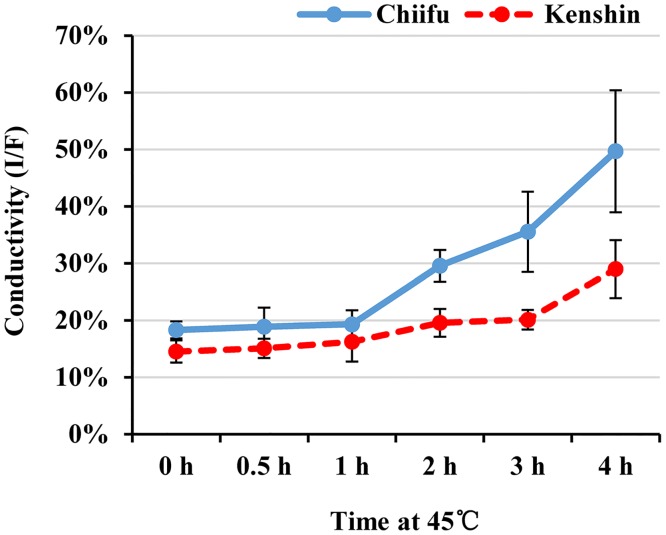
Electrolyte leakage of two DH lines, Chiifu and Kenshin, subjected to treatment at 45°C. Leakage is expressed as the ratio (%) of the conductivity of the initial (I) and final (F) solution. Error bars represent SD of six replicates.

Along with survival percentage, lipid peroxidation, and peroxidase (POD) activity, electrolyte leakage was considered to be a good indicator of thermotolerance in *Brassica* species [40]. Therefore, we measured leakage during HT exposure of two inbred Chinese cabbage lines, Chiifu and Kenshin. As shown in Fig 1, the two lines exhibited similar electrolyte leakage after 1 h exposure to 45°C, but a significant difference was observed after 2 h. On the basis of this result, we concluded that Kenshin is more resistant to > 2 h exposure to HT at 45°C, exactly the reverse of the two strains’ relative tolerance to freezing temperatures [28]. The major parameters used to phenotype thermotolerance of crop plants include viability, pollen development and fertility, photosynthetic rate, and germination [41]. However, breeders and farmers of the leafy vegetable *B*. *rapa* pay much more attention to leaf defense against pathogens, growth, and the heading process, complicating the choice of good marker for thermotolerance. Our results indicate that electrolyte leakage could be a useful marker for temperature sensitivity in *B*. *rapa*.

### Microarray analysis to identify HSR genes

**Fig 2:**
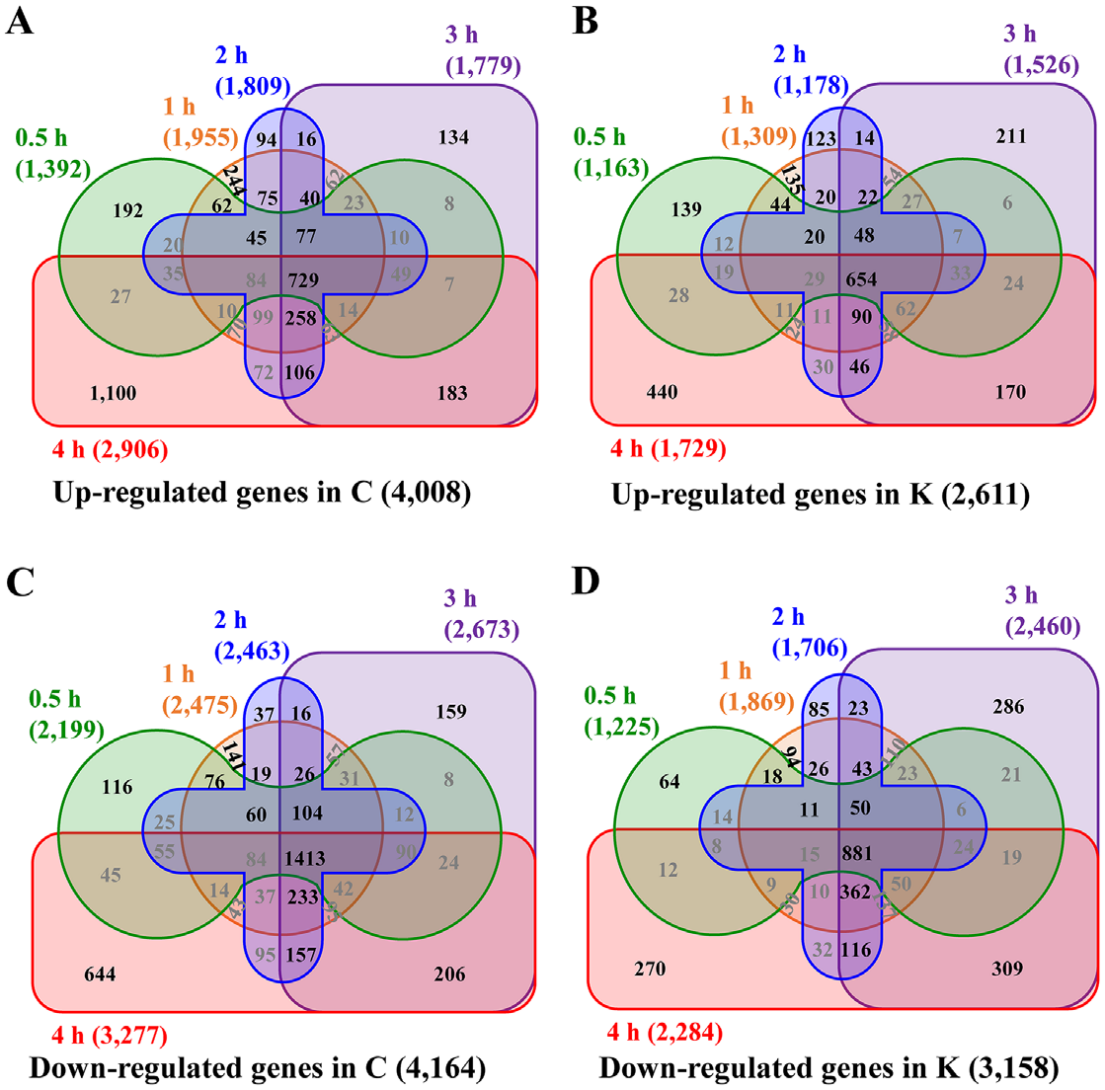
Distribution of genes up- or down-regulated by heat. **A**, Distribution of genes up-regulated by heat in Chiifu; **B**, Distribution of genes up-regulated by heat in Kenshin. Gray number means the stepwise priming up-regulated genes. **C**, Distribution of heat down-regulated genes in Chiifu; **D**, Distribution of heat down-regulated genes in Kenshin. Black numbers indicate the genes down-regulated at a single time point or continuously.

To identify HSR genes, we identified DEGs under HS from among 41,173 genes represented on Br135K microarrays (S3 Table). After removing genes with PI (Probe Intensity) values below 500 for all samples (usually it’s hard to detect the gene expression level using RT-PCR under 25 cycles condition), DEGs were defined as genes with > 2-fold changes in at least one time point. A large number of genes, 4,008 for Chiifu and 2,611 for Kenshin, were up-regulated (Fig 2A and 2B, S4 and S5 Tables). Among these, 729 and 654 genes in Chiifu and Kenshin, respectively, were up-regulated > 2-fold during HS treatments of 0.5–4 h, suggesting that about 20% of HS up-regulated genes (18.2% in Chiifu; 25% in Kenshin) were expressed more strongly during HSR (Fig 2A and 2B, S4 and S5 Tables). In addition, many genes were down-regulated by HS (4,164 in Chiifu and 3,158 in Kenshin) (Fig 2C and 2D, S6 and S7 Tables). Among these, 1,413 and 881 genes in Chiifu and Kenshin, respectively, maintained > 2-fold down-regulation during HS treatments of 0.5–4 h; thus, HS suppressed about 30% of HS-down-regulated genes at all-time points used (Fig 2C and 2D, S6 and S7 Tables). The proportion of DEGs under HS was similar to that obtained in an analysis of the tomato transcriptome (2,203 genes, 9.6%) [13], but higher than the 2% reported by other studies [42–44].

Next, we functionally analyzed HSR genes by GO enrichment using agriGO [35], based on information about *Arabidopsis* homologs. All *Arabidopsis* counterparts of our microarray probes were used as background references, and significantly represented GO items were defined as FDR values below 0.05. At a significance of < 0.05 FDR, 63 and 106 GO items were significantly enriched among the up-regulated genes in Chiifu and Kenshin, respectively (S8 Table). On the other hand, 126 and 145 GO items in Chiifu and Kenshin, respectively, were significantly enriched among the down-regulated genes (S9 Table). The most enriched GO items among the up-regulated genes in both genotypes were associated with HS: response to heat (GO:0009408), response to ROS (GO:0000302), response to temperature stimulus (GO:0009266), response to abiotic stimulus (GO:0009628), MAPKKK cascade (GO:0000165), etc. (S8 Table). Some GO terms exhibited genotype-specific up-regulation: for Chiifu, response to water deprivation (GO:0009414), and for Kenshin, calcium-mediated signaling (GO:0019722), protein targeting to membrane (GO:0006612), and respiratory burst (GO:0045730) (S8 Table).

To determine the association of HSR genes with specific pathways, we placed the *Arabidopsis* counterparts of the identified DEGs on KEGG pathway maps. This analysis identified 126 pathways (S10 Table), including plant hormone signal transduction, starch and sucrose metabolism, plant–pathogen interaction, glutathione metabolism, and so on. Most genes related to the RNA degradation pathway were up-regulated by HS in both Chiifu and Kenshin. Most of genes related to cyanoamino acid metabolism and the *N*-glycan biosynthesis pathways were down-regulated by HS in both Chiifu and Kenshin. We identified no pathway specific for the HS response in Chiifu or Kenshin.

### Intrinsic transcriptome differences between Chiifu and Kenshin prior to HT treatment

**Fig 3:**
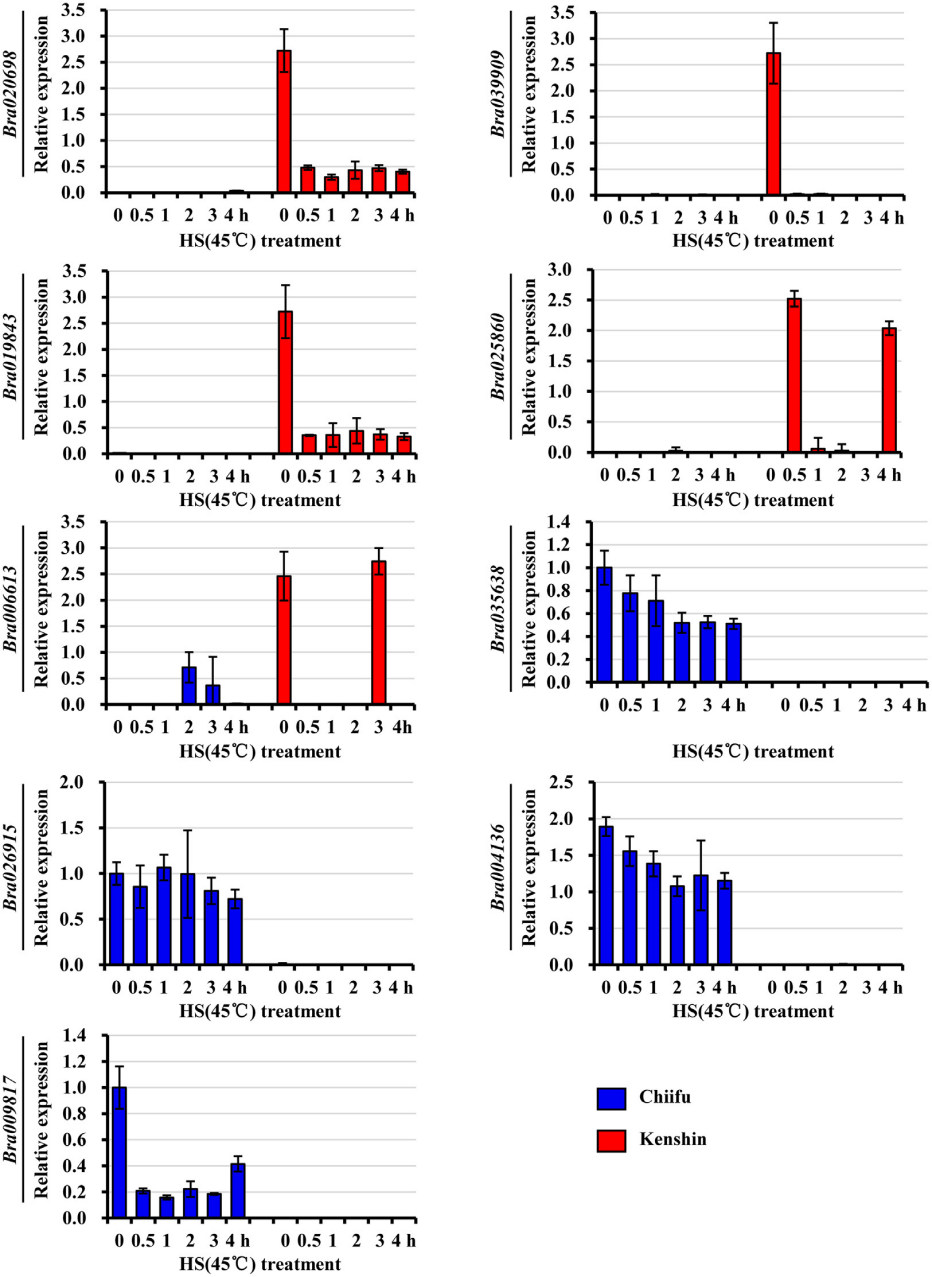
qRT-PCR analysis of several SEGs from either Kenshin or Chiifu. Relative expression was normalized against *BrActin*. Two biological repeats were performed, and error bars represent SD. HS, heat shock.

Because previous reports suggested that plant HS tolerance was largely due to constitutive expression of many genes prior to stress treatment [45,46], we compared the intrinsic differences between two Chinese cabbage inbred lines. Intrinsic genes were selected by fold change on the basis of PI values. A large number of genes, 522 genes for Chiifu and 205 genes for Kenshin (S11 Table), exhibited a > 4-fold change compared to the other, but the functions of most genes exhibiting high levels of expression could not be associated with HS. Many unknown genes (including orphan genes) were in this category, suggesting that they play roles in the HSR. To analyze the expression patterns of SEGs in each inbred line, we selected some of the top-ranking genes from S11 Table and subjected them to qRT-PCR analysis (Fig 3). None of these genes have yet been functionally characterized, but expression of all of them except *Bra026915* was reduced upon HS treatment. To isolate the candidate genes which may work for the heat tolerant in Kenshin, we isolated genes with intrinsically expressed in Kenshin and showed up-regulation in Keshin after heat treatment at the same time. In the end, 14 genes, including one Hsf gene (*Bra029292*, *HSFB2A*), one methionine sulfoxide reductase (*Bra019187*, *PMSR4*), three orphan genes (*Bra039796*, *Bra016685*, and *Bra028606*), were identified.

To obtain functional clues about the genes in this category, we carried out GO annotation on the basis of the *Arabidopsis* homolog data (Table 1). Genotypic SEGs included those encoding kinases, TFs, and factors involved in response to stress, response to abiotic or biotic stimulus, transferase activity, hydrolase activity, and signal transduction. Noteworthy categories include cellular respiration, unsaturated fatty acid biosynthesis, and Hsf genes, which were over-represented in Kenshin relative to Chiifu. For instance, 5 Hsf genes, *Bra013409*, *Bra029292* (*HSFB2A*), *Bra029291* (*HSFB2A*), *Bra004272* (*HSFA8*), and *Bra000557* (*HSFA2*) were highly intrinsic expressed in Kenshin. Otherwise, only one Hsf gene, *Bra012828* (*HSFA7A*), showed highly intrinsic expressed in Chiifu. We also carried out KEGG pathway analysis to reveal differences between the two genotypes (S12 Table). In total, 100 and 86 pathways were represented by the up-regulated genes in Chiifu and Kenshin, respectively. Metabolic pathways were most prominent in both genotypes, suggesting that a metabolomics approach will be required in future studies.

**Table 1:** GO annotation of intrinsically expressed genes. One gene can be associated with two or more GO terms. NA, no *Arabidopsis* homologs.

GO Term	Kenshin-up	Chiifu-up
**Kinase**	173	322
**Response to stress**	167	320
**Response to abiotic or biotic stimulus**	154	302
**Transferase activity**	110	220
**Hydrolase activity**	110	189
**Signal transduction**	66	161
**Transcription factors**	61	103
**Organelle membrane**	70	61
**Vacuole**	60	53
**Zinc ion binding**	39	71
**Oxidation-reduction process**	39	67
**Response to salicylic acid stimulus**	22	71
**Carbohydrate catabolic process**	38	31
**Response to water deprivation**	25	43
**Response to ethylene stimulus**	19	43
**Response to auxin stimulus**	17	30
**Response to heat**	13	29
**Photosynthetic membrane**	24	16
**Chlorophyll binding/metabolic process**	14	12
**Photosynthesis**	14	10
**Heat acclimation**	3	15
**Heat shock protein**	5	13
**Proline transport**	10	6
**Respiration**	9	7
**Water transport**	8	7
**Sugar transmembrane transporter activity**	5	8
**Cellular respiration**	8	2
**Unsaturated fatty acid biosynthetic process**	6	2
**Heat shock transcription factor**	5	1
**Unclassified**	292	465
**NA**	203	196

### Analysis of some HSR genes

**Fig 4:**
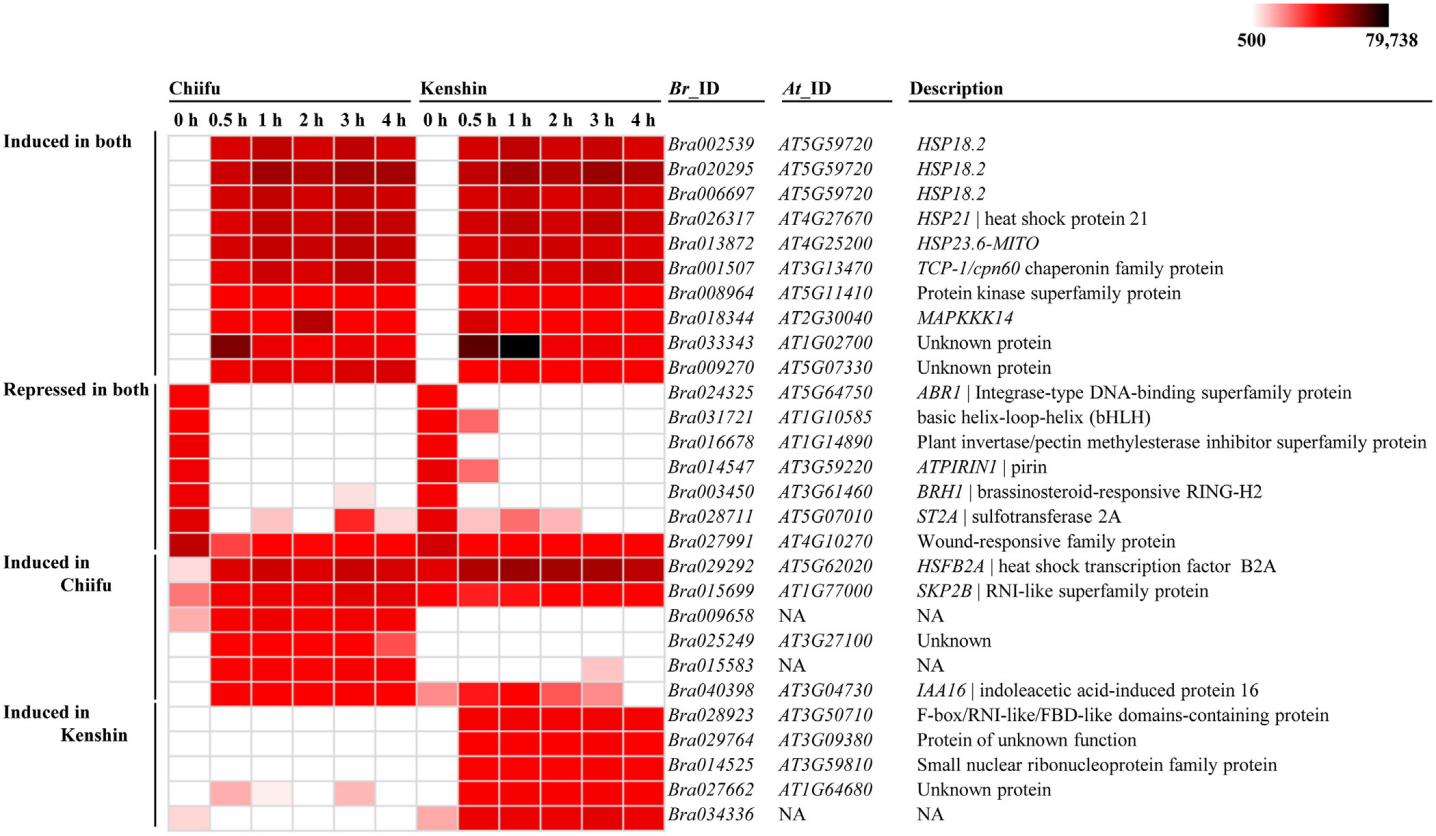
Heat map of genes induced/repressed by HS treatment.

As a first step toward identifying putative regulatory genes for thermotolerance, we identified several genes exhibiting very high levels of expression or specific expression changes, and confirmed them by RT-PCR (Fig 4, S1 Fig, and S13 Table). The top ten genes induced by HS in both genotypes included genes encoding small Hsps, chaperones, kinases, and unknown proteins. Many of these are well-known genes involved in thermotolerance in plants [3,19,47]. Among these, *Bra002538* (*HSP18*.*2*) exhibited the strongest induction in both genotypes, > 410-fold at 0.5 h and > 630-fold at 1 h, and maintained its level thereafter. An unknown gene (*Bra033343*, *AT1G02700*) exhibited a peculiar pattern of expression upon HS treatments: in Chiifu, 1,213-fold induction at 0.5 h and 217-fold induction at 1 h, but in Kenshin, 1,573-fold at 0.5 h, 2,462-fold at 1 h, and 176-fold at 2 h. These patterns are similar to *Hsp90* and *Hsp70* expression, as expected for the HSR. Several genes were specifically induced in either genotype. Among them, Kenshin SEGs (*Bra028923*, *Bra029764*, *Bra014525*, *Bra027662*, and *Bra034336*) could be good targets for thermotolerance studies in Chinese cabbage because Kenshin has been traditionally used as a breeding stock to develop heat-tolerant plants [22,23].

### Hsfs and Hsps

Many Hsfs and Hsps play important roles in plant heat tolerance. In the Br135K microarray, 38 Hsf- and 206 Hsp-encoding genes (*Hsp100/ClpB*, *Hsp90*, *Hsp70*, *Hsp60*, *Hsp40*, and *sHsp*) are represented (S14 Table). Among them, 49 genes (including 11 *Hsf* genes) exhibited very low levels of expression (PI values < 500 of in all samples). Upon 1 h HS treatment, however, 10 and 13 *Hsf* genes were up-regulated > 2-fold in Chiifu and Kenshin, respectively. Among these, nine *Hsf* genes were up-regulated in both genotypes: *HsfA2* (*Bra000557*), *HsfA4A* (*Bra020988*), *HsfA7A* (*Bra012828*, *Bra033468*), *HsfB2A* (*Bra029292*, *Bra029291*, *Bra010049*), *HsfB2B* (*Bra000749*), and *HsfB3* (*Bra000235*). Up-regulated expression of some genes was confirmed by RT-PCR (S2 Fig). In particular, one *HsfA2* and two *HsfA7A*s were up-regulated over 4-fold in both genotypes, whereas one *HsfB2A* (*Bra029292*) was induced over 20-fold only in Chiifu. The expression levels of three *HsfB2A*s in Kenshin (*Bra010049*, *Bra029291*, & *Bra029292*) were relatively high under normal growth conditions and increased further upon HS treatment, whereas the levels of two *HsfB2A*s in Chiifu (*Bra029291*, *Bra010049*) exhibited similar patterns upon HS treatment but were lower than those in Kenshin. This result suggests that *BrHsfB2A* (*Bra029292*) plays an important regulatory role in heat tolerance in Kenshin. In tomato, *HsfA1a*, *HsfA3*, *HsfB1*, and *HsfB2a* are most abundant and exhibit only minor changes in response to heat. On the other hand, expression of low-abundance genes, such as *HsfA1b*, *HsfA1e*, *HsfA2*, *HsfA4b*, and *HsfA6a*, increase upon HS treatment [13]. Some Chinese cabbage *Hsfs* (*HsfB1*, *HsfB2A*) exhibited expression profiles similar to those observed in tomato, whereas others did not, indicating the presence of distinct regulatory mechanisms in different plant species.

The *Arabidopsis* genome contains 21 *Hsf* genes that can be categorized into three classes (A, B, and C) [10,48]. HsfA1a is a master regulator of the HSR [10,12,13,49]. However, given the low expression level of *HsfA1* following HS, our data imply that other Hsfs could serve as the major TFs in *B*. *rapa*. *HsfA4a* and *HsfA8* are sensors of reactive oxygen species (ROS), which are produced as a secondary stress signal during the HSR [50,51]. In particular, HsfA4 acts as a sensor of the H_2_O_2_ signal [15], whereas *HsfA5* acts as a negative regulator of this pathway [16]. In our microarrays, both genes were highly expressed. Although the expression of *HsfA4* increased slightly upon HS, the expression of *HsfA5* decreased slightly (S14 Table). All these results suggest that heat tolerance in *B*. *rapa* might be primarily regulated at the protein level, with only minor regulation at the transcriptional level. Alternatively, heat tolerance might involve key regulators that are different from those *Arabidopsis*, as proposed in Wang et al. [52].

Like Hsfs, Hsps also play important roles in heat tolerance in plants, especially in protein folding, assembly, translocation, and degradation [17]. In our microarrays (S14 Table), most *Hsp70*, *Hsp90*, and *Hsp101* genes were constitutively and highly expressed in all samples. However, small Hsps encoded by genes such as *Hsp18*.*2*, *Hsp20*, *Hsp22*, and *Hsp23*.*6* exhibited strong induction (15–656-fold) by HS treatment in both genotypes. We confirmed the expression of some genes by RT-PCR (S2 Fig). These results are similar to those in tomato, in which the majority of HS-induced chaperone genes belong to the sHsp family [13].

In *Arabidopsis*, HS-associated 32 kD protein (HSA32) (AT4G21320), an Hsp, is essential for acquired thermotolerance during long-term recovery after acclimation treatment [53] and in rice [54]. *B*. *rapa* does not contain ortholog of this gene, providing further evidence for the existence of different HSRs among plants. Recently, Wang et al. [52] reported that different key genes and regulatory mechanisms are involved in abiotic stress responses in *B*. *rapa* and *Arabidopsis*.

### Upstream components of the HSR, including ROS

Upstream events of the HSR include perception and signal transduction of HS. Jia et al. [55] demonstrated that Hsf activity and Hsp production involved in thermotolerance are a result of cross-talk among H_2_O_2_, nitric oxide (NO), Ca^2+^ channels, and calmodulin (CaM). In particular, CaM activates Hsfs, protein kinase, phosphatase, and cyclic nucleotide-gated ion channels. Among these, ROS including H_2_O_2_ are considered to be the first signaling components produced by HS [15,56,57]. The ROS signal can then be recognized by histidine kinases and Hsfs (HsfA4 and HsfA8) [19,50,51]. Hsf sensing activates multiple TFs (Zat family, WRKY, and MBF1c) through the MAPK signal pathway [19].

Regarding the expression of ROS-scavenging genes upon initiation of the HSR, two possibilities exist: such genes could be induced in order to reduce levels of ROS produced by HS, or they could be suppressed in order to maintain the levels of ROS involved in downstream signaling. Among 170 PODs on the Br135K microarray, only seven were differentially expressed upon HS treatment (S15 Table). Three genes were up-regulated in both genotypes: *ascorbate peroxidase 1* (*APX1*) (*Bra031598*), *Fe-superoxide dismutase 3* (*FSD3*) (*Bra026503*), and *Fe-superoxide dismutase 2* (*FSD2*) (*Bra029190*). Previous work revealed *APX1* as a central component of the ROS gene network [58] and a key player in stress responses [59], whereas *FSD2* and *FSD3* confer protection against oxidative stress [60,61]. *Glutathione peroxidase 2* (*GPX2*) (AT2G31570; *Bra022853*), which exhibits Kenshin-specific up-regulation, protects against stress through activation of cytosolic *superoxide dismutase* [61]. Thus, *BrGPX2* is a promising candidate thermotolerance-related gene in Kenshin. In addition, one peroxidase superfamily protein (*Bra016930*), which is also likely to be involved in ROS signaling, was down-regulated upon HS in both genotypes.

Because calcium signaling can engage in cross-talk with ROS signaling to trigger thermotolerance, we investigated the expression profiles of several calcium-related genes represented on the Br135K chip: 24 calcium channels, 39 CaM, 31 calcium-dependent protein kinases (CDPKs), five CaM-binding protein phosphatases, and 33 cyclic nucleotide-gated ion channels (*CNGCs*) genes (S16 Table). Most calcium channel genes were highly expressed at all-time points, suggesting that they are regulated at the protein level, if at all. In contrast to the nine *Arabidopsis CaM* genes up-regulated by HS treatment [62], 14 *BrCaM* genes (with the exception of *CaM8*) exhibited high constitutive expression under most conditions. These data further support the idea that the HSR is differentially regulated among species. Expression of CDPK and CNGC genes exhibited no significant changes after HS treatment, with the exception of *Bra001676*, a homolog of *Arabidopsis* salt-induced *CNG20* [63], which had Kenshin-specific expression. Taken together, these data suggest that regulation of calcium-related proteins in *B*. *rapa* HSR is exerted at the protein level.

ROS signaling recognized by histidine kinases must be transmitted to other kinases and phosphatases. To obtain further insight into the HSR signaling pathway, we analyzed expression profiles of 2,110 kinase genes represented on the Br135K microarray. Several genes exhibited differential expression in response to HS treatment: in total, we detected 13 up-regulated, three down-regulated, five Kenshin-specific, and seven Chiifu-specific genes (S17 Table). The 13 up-regulated genes included receptor kinase 3 (*RK3*) (*Bra001630*, *Bra040589*, *Bra038767*, *Bra040589*), mitogen-activated protein kinase kinase kinase 14 (*MAPKKK14*) (*Bra018344*), a protein kinase superfamily (*AT2G28940*; *Bra040931*) (AT5G11410; *Bra008964*) (*AT5G13290*; *Bra008839*), a U-box domain-containing protein kinase family (*Bra024393*), and *somatic embryogenesis receptor-like kinase 5* (*Bra032128*). The three down-regulated genes included *CBL-interacting protein kinase 13* (*Bra021897*), a receptor-like protein kinase-related family (*AT5G48540*; *Bra037480*), and a leucine-rich repeat protein kinase family (*AT1G51800*; *Bra030416*). The five Kenshin-specific genes included *with-no-lysine (K) kinase 4* (*Bra024518*), a protein kinase superfamily (*AT3G26700*; *Bra034245*), a concanavalin A-like lectin protein kinase family (*Bra010340*), a *cysteine-rich receptor-like protein kinase* (*RLK*) *36* (*Bra029496*), and a leucine-rich repeat protein kinase family (*AT5G59680*; *Bra020301*). Finally, the seven Chiifu-specific genes included *wall-associated kinase 5* (*Bra025886*), *phosphoglycerate kinase 1* (*Bra034729*), receptor serine/threonine kinase (*AT4G18250*; *Bra008687*), a leucine-rich repeat protein kinase family protein (AT5G59670; *Bra006700*), a *cysteine-rich RLK 39* (*Bra017552*), and a protein kinase superfamily (*AT3G46930*; *Bra018183*). High expression of most kinase genes under all conditions suggested that these factors are primarily regulated at the protein level.

Although most phosphatase genes were highly expressed in all samples (S18 Table), three were differentially expressed: two were up-regulated genes upon HS (*AT3G51470*; *Bra036796*, *AT4G29690*; *Bra011129*), and one was expressed specifically in Kenshin (probable apyrase 5, *Bra019669*). The functions of these genes have not yet been determined. As with the kinases, the expression patterns suggest that most phosphatases function at the post-transcriptional level.

### Transcription factors (TFs)

TFs regulate many genes involved in plant growth and development at the transcriptional level. The Br135K microarray includes 3,354 TFs (8% of the total of 41,174 genes), of over 43 different classes (Table 2, S19 Table). Among them, 109 TFs exhibited significant changes in their expression levels following HS treatment (Table 2). Apart from the heat-induced Hsfs, the majority of these TFs belong to the Integrase-type, NAC, homeodomain, HB, bHLH, and DREB families. Members of these TF families are involved in the HSR and thermotolerance in different plant species [45,64,65], indicating the existence of complex transcriptional regulatory networks beyond the direct regulatory control of Hsfs.

**Table 2:** Summary of transcription factor genes represented on the Br135K microarray.

		Up-regulated by HS			Down-regulated by HS			Genotype specific	
	No	Chiifu	Kenshin	Both	Chiifu	Kenshin	Both	Chiifu	Kenshin
**MYB**	289	1	-	3	-	-	1	-	-
**bHLH**	234	-	-	1	-	-	4	-	-
**NAC**	191	-	-	7	-	-	3	-	-
**Homeodomain**	183	-	-	3	1	-	1	1	-
**C2H2**	159	-	-	1	-	-	2	1	-
**Integrase-type**	153	-	-	9	-	-	6	-	-
**WRKY**	150	1	-	-	-	-	1	-	-
**AGL**	109	1	-	-	-	-	2	-	-
**bZIP**	108	-	-	1	-	-	-	1	-
**AP2**	100	-	1	-	-	-	-	-	-
**HB**	93	-	-	2	-	-	5	3	-
**GATA**	69	-	-	-	-	-	1	-	-
**Dof**	66	-	-	-	-	-	-	-	-
**MADS**	62	-	-	-	-	-	-	1	-
**B3 family**	59	-	-	-	-	-	-	1	-
**NF-Y**	51	-	-	1	-	-	-	-	-
**C3HC4**	50	-	-	2	-	-	-	-	-
**C-x8-C-x5-C-x3**	43	-	-	-	-	-	3	-	1
**DHHC**	42	-	-	-	-	-	1	1	-
**CCCH**	41	-	-	2	-	-	1	-	-
**LOB**	41	-	-	-	-	-	1	-	-
**HS**	38	-	-	6	-	-	-	-	-
**ERF**	38	-	-	1	-	-	-	-	-
**ARF**	36	-	-	1	-	-	2	-	-
**GRAS**	34	-	-	-	1	-	-	-	-
**TCP**	33	-	-	-	-	-	-	-	-
**B-box**	32	-	-	1	-	-	2	-	-
**Squamosa**	29	-	-	-	-	-	2	-	-
**DREB (TINY)**	28	-	-	5	-	-	1	-	-
**JAZ**	26	-	-	1	-	-	-	-	-
**LSH**	26	-	-	4	-	-	-	-	1
**A20/AN1**	24	-		-	-	-	-	-	-
**PLATZ**	23	-	-	-	-	-	-	-	-
**RWP-RK**	23	-	-	-	-	-	-	-	-
**Jumonji**	22	-		1	-	-	-	-	-
**CCHC**	22	-	-	-	-	-	-	-	-
**ZFP**	20	-	-	-	-	-	-	-	-
**GRF**	17	-	-	-	-	-	-	-	-
**Alfin-like**	17								
**KNAT**	16	-	-	-	-	-	-	1	-
**BZR1 family**	16	-	-	-	-	-	-	-	-
**Winged-helix**	12	-	-	-	-	-	-	-	-
**AUX/IAA**	8	-	-	-	-	-	-	-	-
**OTHER**	521	3	-	6	1	-	10	-	-
**Total**	**3,354**	**3**	**1**	**52**	**2**	**0**	**39**	**10**	**2**

Although only *AP2/B3*-like TF (*AT5G60142*) (*Bra002509*) exhibited Kenshin-specific up-regulation, Chiifu-specific up-regulation was detected for three genes: *MYB-like 2* (AT1G71030) (*Bra016164*), *WRKY33* (*Bra000064*), and *AGL64* (*Bra032347*). Zinc-finger CCCH domain-containing-protein (*Bra012068*) and *LSH6* (*Bra031552*) were Kenshin-specifically expressed without much change upon HS, and ten genes were specific to Chiifu. Except for *WRKY33*, which may play a role in defense signaling, the functions of these genes have not been determined.

According to a recent study, the ER membrane-associated basic leucine zipper (bZIP) transcription factor bZIP28 is activated by ER stress resulting from the accumulation of misfolded or unfolded proteins following HS; this factor stimulates expression of stress response genes [66]. Two *BrbZIP28* genes were highly expressed in all Chinese cabbage samples; expression of one of them (*Bra034147*) increased slightly upon HS treatment, whereas the other gene (*Bra023224*) exhibited no significant change.

**Fig 5:**
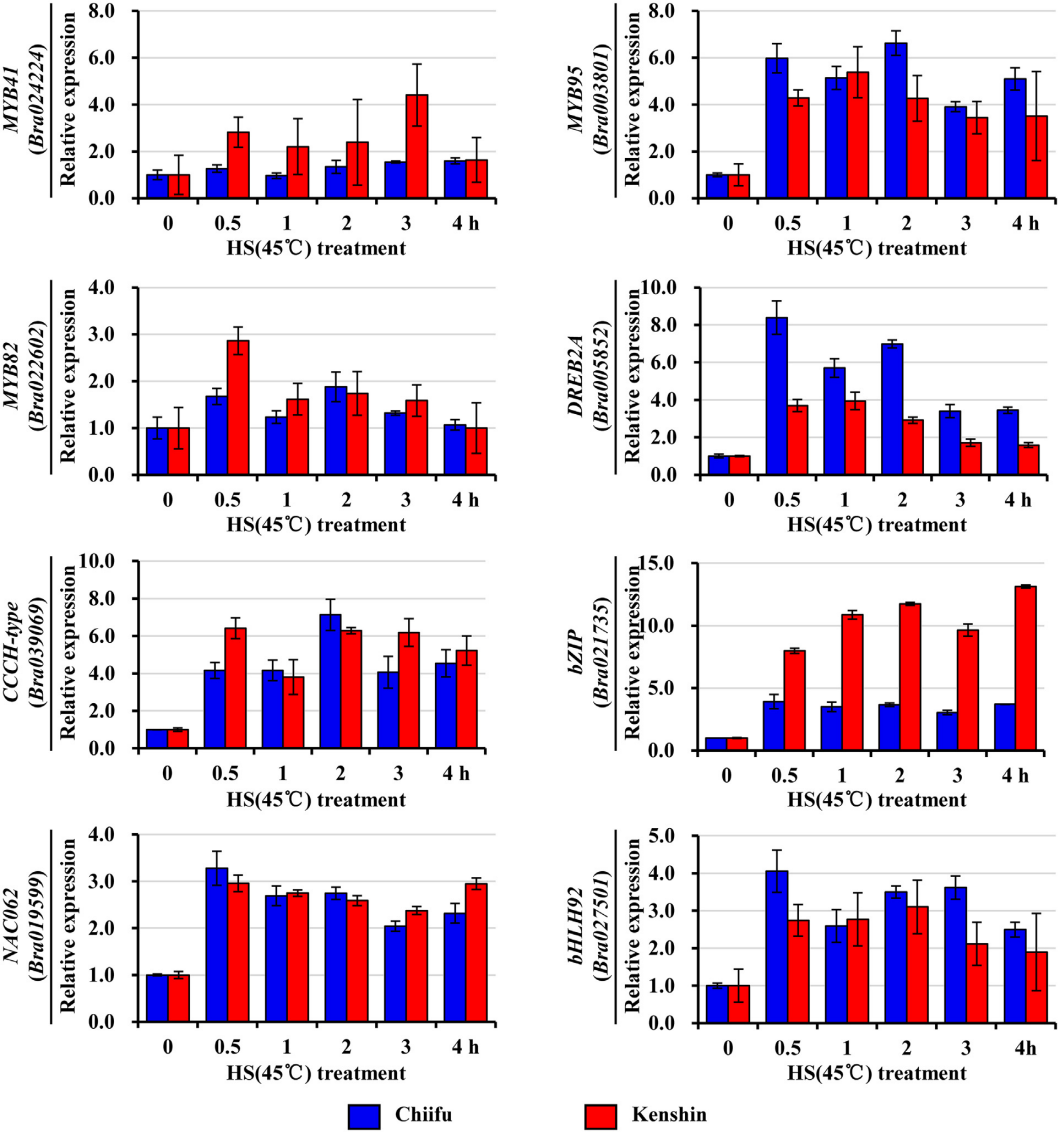
qRT-PCR analysis of transcription factor genes that were up-regulated by HS treatment. Relative expression was normalized to *BrActin* and is presented relative to 0 h expression levels. Two biological repeats were performed, and error bars represent SD. HS, heat shock.

Several TFs were selected and subjected to qRT-PCR analysis. Three genes (*Bra022602*, *Bra039069*, *Bra019599*) were induced in both lines at similar levels upon HS, and three and two genes exhibited prominent expression in Chiifu (*Bra003801*, *Bra005852*, *Bra027501*) and Kenshin (*Bra021735*, *Bra024224*), respectively (Fig 5). Due to the large standard deviation resulting from normalization, the qRT-PCR data of several genes (*MYB41*, *MYB95*, *MYB82*, and *bHLH92*) were not consistent with the microarray values. Except for *Bra022602* (*MYB82*), expression of most genes was highly increased by HS treatment. With the exception of three genes (*CCH-type*, *MYB82*, and *MYB95*) whose functions have not been identified, these genes are related to abiotic stress responses in *Arabidopsis*. Two genes with prominent expression in Chiifu, *Bra005852* (*DREB2A*: dehydration-responsive element binding protein 2A) and *Bra027501* (*bHLH92*), are responsive to cold stress [28] suggesting that they serve another role in HSR. In *Arabidopsis*, bHLH92 functions in response to NaCl, dehydration, and cold [67], but its homolog, *Bra027501*, would be expected to function in HS of *B*. *rapa*, based on its expression change. *Arabidopsis* DREB2A controls the levels of HsfA3, Hsp18.1-CI, Hsp26.5-MII, and Hsp70 during heat and drought stress [68–70], implying that it responds to both heat and cold stresses. *B*. *rapa* contains three *DREB2A* genes: two of these (*Bra005852*, *Bra019162*) were expressed at low basal levels under normal growth conditions and were greatly up-regulated by HS, whereas the other (*Bra009112*) was expressed at a relatively high level that increased further upon HS treatment (S19 Table). These results indicate that expression of Hsps in *B*. *rapa* might be also induced by DREB2A.

Two genes exhibiting a similar response to HS treatment in both inbred lines, *Bra003801* (*MYB95*) and *Bra019599* (*NAC062*/*NTL6*), are predicted to function in both cold and HT, even though only the cold response has been characterized in *Arabidopsis* [71,72]. In *Arabidopsis*, NAC062/NTL6 processing is stimulated by cold and ABA to activate the expression of defense response genes, but in *B*. *rapa*, its processing is reduced by HT [71]. Given that the expression of *Bra019599* was greatly increased upon HS in both lines, it is possible that *BrNAC62* functions in both cold and HT stress.

Two genes with a notable response to HS treatment in Kenshin, *Bra024224* (*MYB41*) and *Bra021735* (a bZIP/*AIR1* [*Anthocyanin-Impaired-Response-1*]), may function in HS tolerance. *Arabidopsis* MYB41 activates suberin synthesis under abiotic stress conditions [73], implying a relationship to HS. *Arabidopsis AIR1* regulates various steps in the flavonoid and anthocyanin accumulation pathway [74]. Its expression is regulated by salt stress, but not by HT or drought. It is possible that *BrAIR1* could function under HS conditions, particularly for Kenshin. Therefore, *BrAIR1* could be a useful molecular marker for HT tolerance in *B*. *rapa*.

HSR genes and proteins can be divided into two groups, signaling components (protein kinases and TFs) and functional genes (HSPs and catalase) [75,76]. Zat7 [77], Zat10 [15], and Zat12 [78] respond to HS. In particular, Zat12 is necessary for the expression of *APX*, *Zat7*, and *WARKY 25*. *B*. *rapa* contains *Zat1*, *6*, *10*, and *12*, but not *Zat7*. The expression levels of *Zat12* (four alleles) and *Zat10* (two alleles) were very high and slightly increased by HS. Also, *WRKY25* expression was very high. These results suggest the possible involvement of TFs through the MAPK signaling pathway.

### HS marker genes

**Fig 6:**
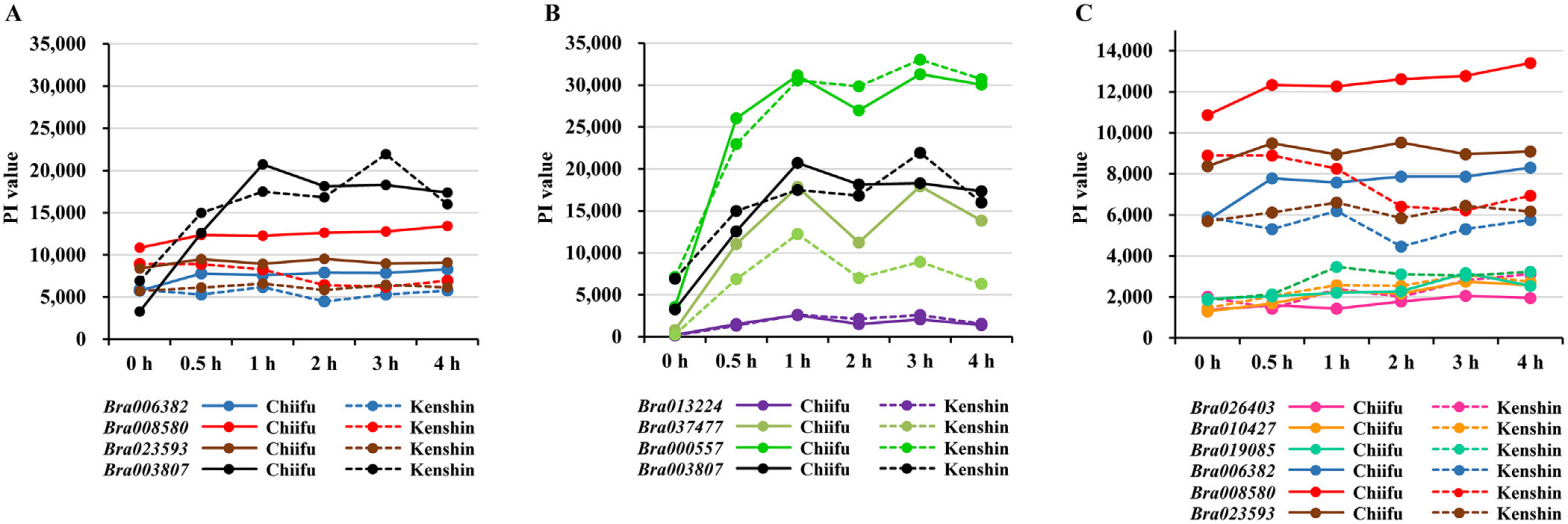
Expression patterns of marker genes for thermotolerance in Chiifu and Kenshin. PI value indicates probe intensity (see also S20 Table). **A**, Expression patterns of basal thermotolerance marker genes (BT) and short-term acquired thermotolerance (SAT) in Chiifu and Kenshin. **B**, Expression patterns of long-term acquired thermotolerance (LAT) marker genes in Chiifu and Kenshin. **C**, Expression patterns of thermotolerance to moderately high temperatures (TMHT) marker genes in Chiifu and Kenshin.

According to a previous report [41], *Arabidopsis* plants exhibit four classes of tolerance to HS regimes: basal thermotolerance (BT), short-term acquired thermotolerance (SAT), long-term acquired thermotolerance (LAT), and thermotolerance to moderately high temperature (TMHT). Because genes representing each regime have been identified in *Arabidopsis*, we examined expression of the *B*. *rapa* genes that correspond to the *Arabidopsis* genes involved in the tolerance phenotypes (S20 Table, Fig 6). Expression of most genes did not differ between Chiifu and Kenshin, but only expression of *XPO1A* (*exportin 1A*) (*Bra008580*, *Bra006382*), which is involved in BT and TMHT in *Arabidopsis*, seemed to be correlated with thermotolerance as well as membrane leakage. LAT-related genes could be up-regulated (induced or stimulated) even after 30 min HT treatments, but no difference was observed between the two genotypes. The *Hsa32* (*HS-associated 32 kD*: *AT4G21320*) homolog, which is also a marker of LAT, was absent from the *B*. *rapa* gene database. These results suggest that (1) HSR markers differ between *Arabidopsis* [41] and *B*. *rapa*, and (2) BT and LAT from *B*. *rapa* are controlled by different genes.

### Membrane leakage-related (MLR) genes

**Fig 7:**
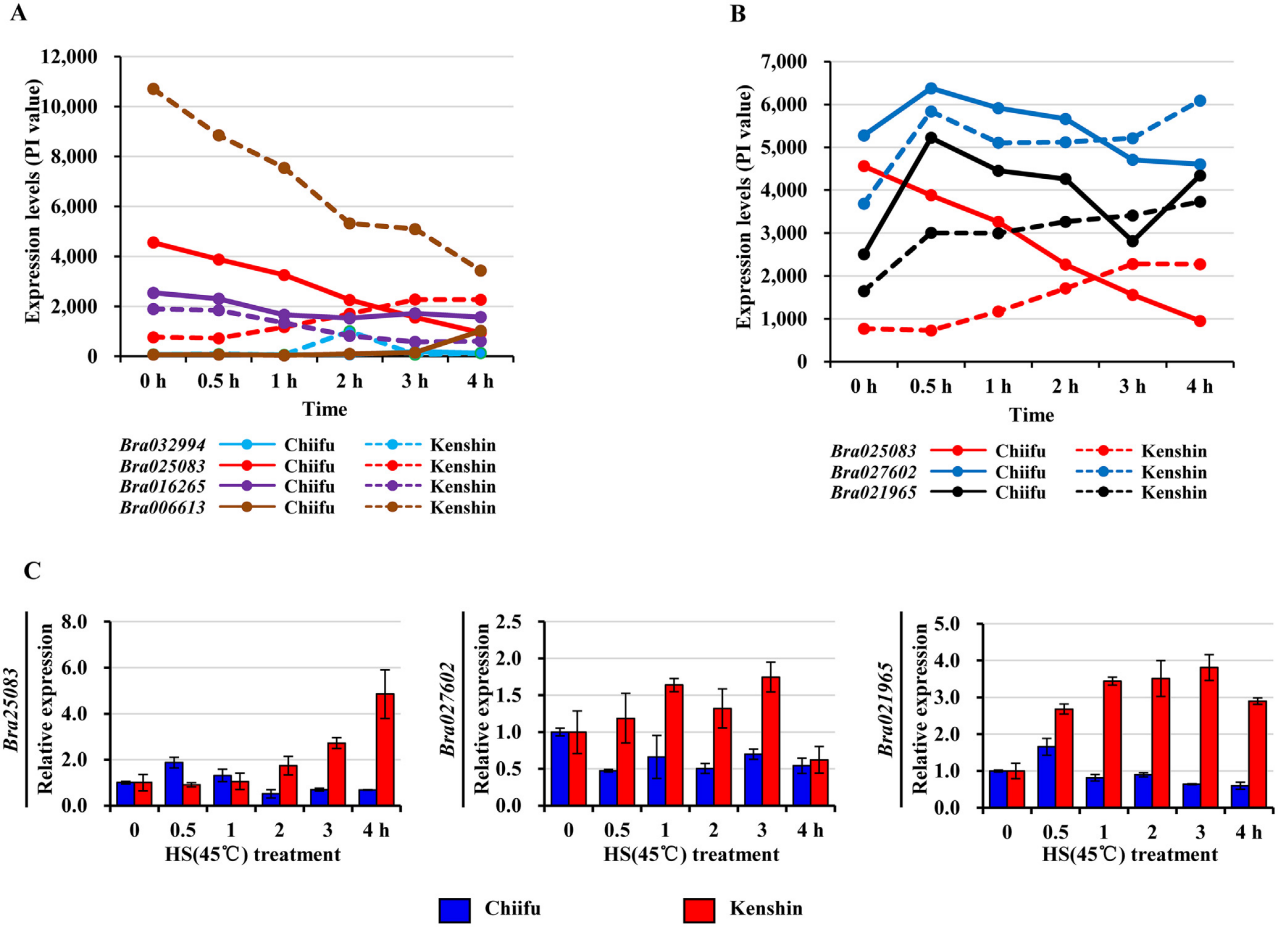
Examples of membrane leakage-related (MLR) genes. A, Four genes: two up-regulated genes, *Bra032994* (NA) and *Bra025083* (*cytochrome P450*; *CYP707A3*), and two down-regulated genes, *Bra006613* (*FAD*/*NAD(P)-binding oxidoreductase*) and *Bra016265* (*protein kinase superfamily protein*). B, Three *B*. *rapa* genes for *CYP707A3*. C, qRT-PCR of three *B*. *rapa* cytochrome P450 genes (*abscisic acid 8’-hydroxylase*). Relative expression was normalized to *BrActin* and is presented relative to 0 h expression levels. Two biological repeats were performed and error bars represent SD. HS, heat shock.

Even though HSR genes were immediately induced by HT exposure, membrane leakage of *B*. *rapa* remained constant until 1 h HT exposure (Fig 1). Regarding electrolyte leakage, Kenshin is less sensitive to prolonged HT exposure than Chiifu, implying that the HT responses of these lines are negatively correlated with leakage. Because electrolyte leakage reflects the traits of these two inbred lines, analysis of the MLR genes is important for an understanding of the *B*. *rapa* HT-response. MLR genes can be defined as those whose expression patterns correlate with the membrane leakage pattern either positively or negatively, i.e., expression of genes should not exhibit a significant change from no treatment to 1 h HS treatment, but should start to change upon 2 h HS treatment (over 2-fold, up or down) and increase thereafter. As shown in S21 Table, expression levels of 15 and 65 genes were changed over 2-fold in Chiifu and Kenshin, respectively. Among them, four genes specifically expressed in Kenshin were selected as putative MLR genes: two up-regulated genes [*Bra032994* (NA), *Bra025083* (cytochrome P450; *CYP707A3*)], and two down-regulated genes [*Bra006613* (FAD/NAD(P)-binding oxidoreductase) and *Bra016265* (protein kinase superfamily protein)](Fig 7A and 7B). In particular, the *Bra025083* expression pattern was well matched to the membrane leakage pattern. *B*. *rapa* includes three *CYP707A3* genes, and two of them, *Bra025083* and *Bra021965*, exactly followed the pattern of membrane leakage (Fig 7C). The reported functions of CYP707A3, a major ABA 8’-hydroxylase, includes the dehydration response [79,80], stomata closure [81], and salt stress [80]. Our data suggests another function for this gene in HS conditions.

### Orphan genes

**Fig 8:**
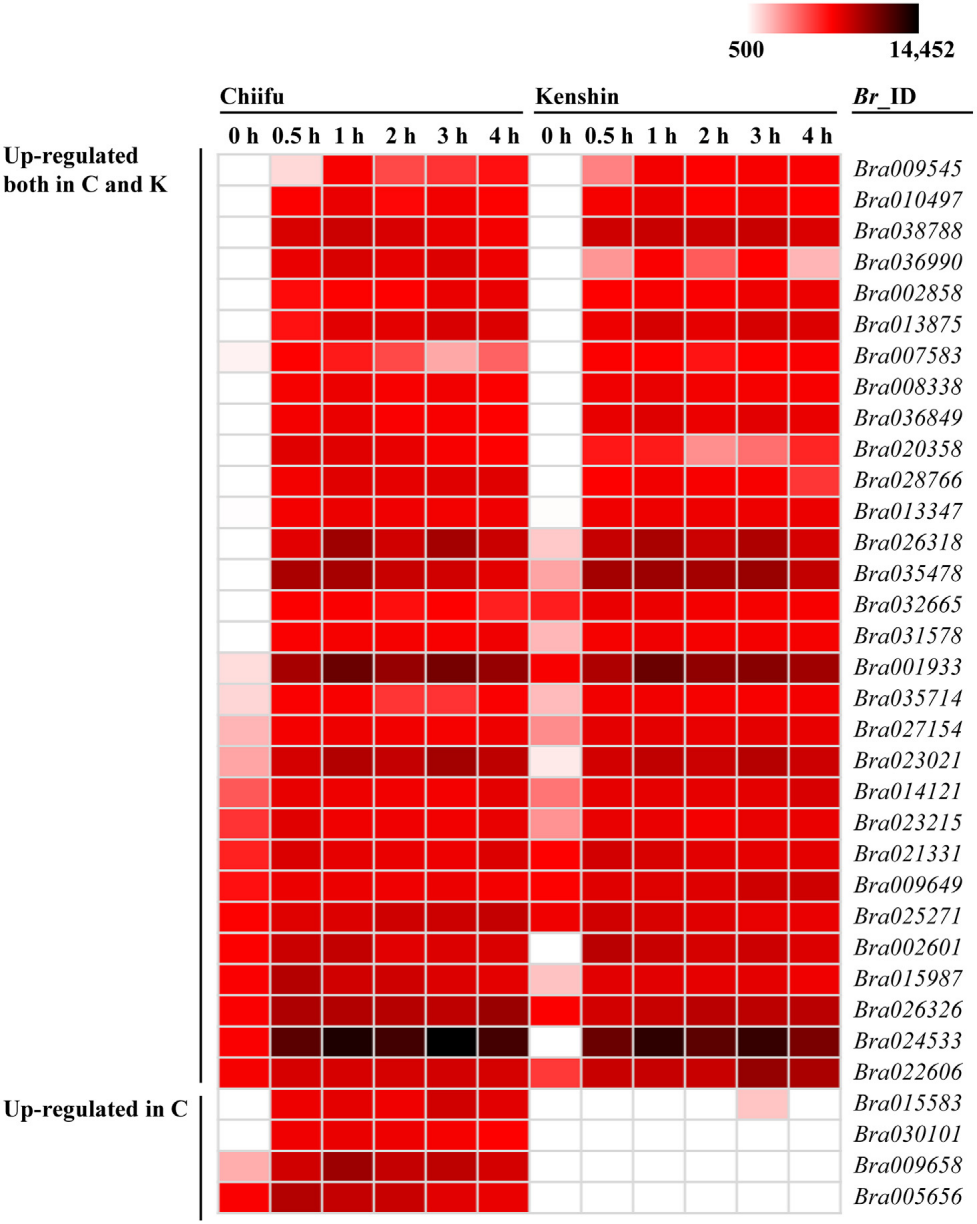
Heat map of orphan genes induced by HS treatment.

**Fig 9:**
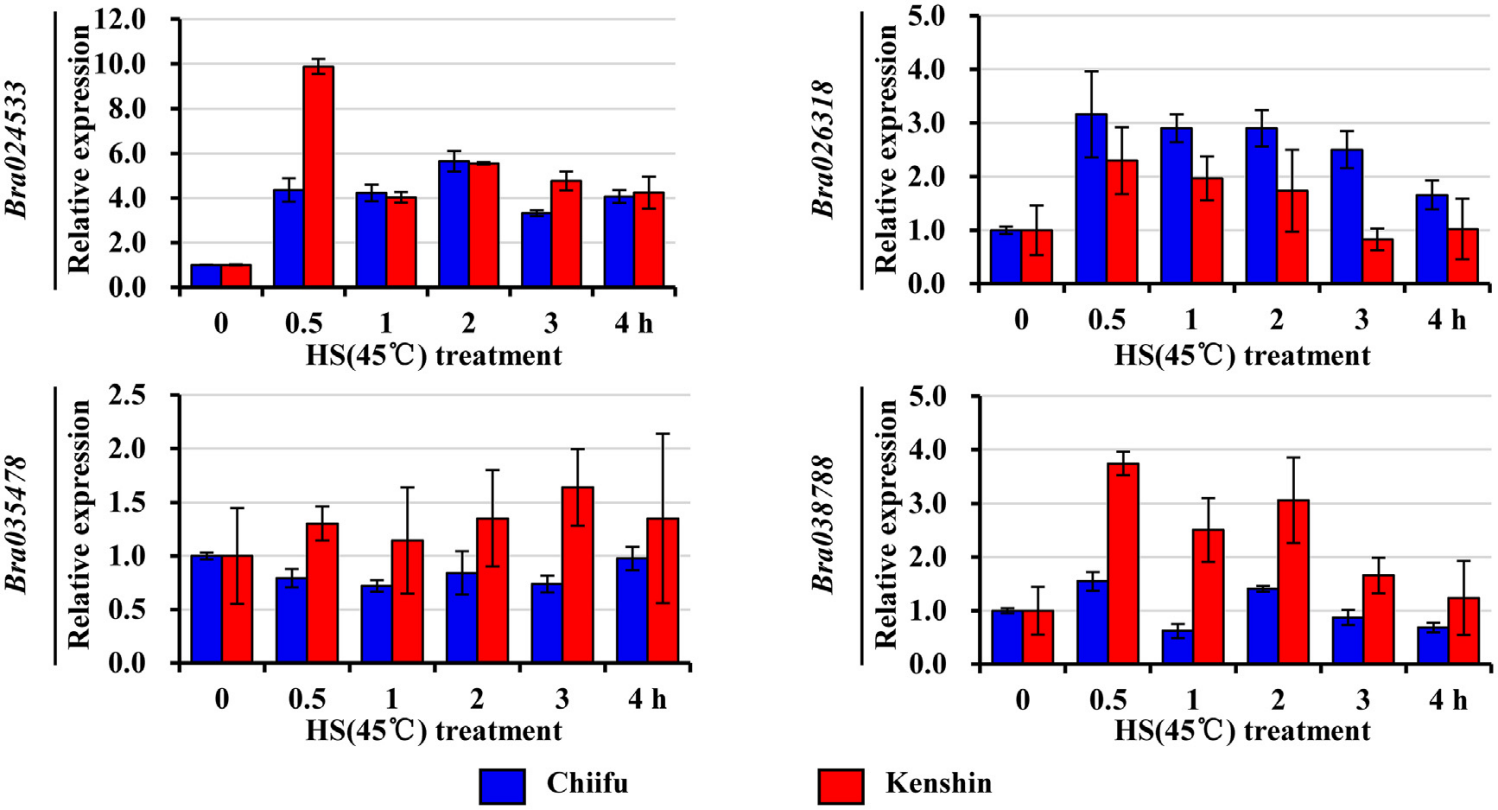
qRT-PCR verifications of the expression of several orphan genes. Relative expression was normalized to *BrActin* and is presented relative to 0 h expression levels. Two biological repeats were performed, and error bars represent SD. HS, heat shock.

Orphan genes, which are protein-coding genes unique to a species, are widespread across all organisms [82]. Estimates of the percentage of orphan genes in various species range from 1% to 71%, with 5–15% being fairly typical [83]. Orphan genes are associated with abiotic stresses, including HS [83–85]. About 13% of genes on the Br135K microarray were orphan genes (5,349 out of 41,173 genes) (S22 Table). Among them, 34 were induced or up-regulated by HS treatment, and 16 and 13 genes were induced in a Chiifu- or Kenshin-specific manner (S22 Table, Fig 8). *Bra026318* (15–37-fold induction) and *Bra024533* (25–34-fold induction) exhibited the strongest induction in Chiifu and Kenshin, respectively. In addition, 54 and 14 genes were specific to Chiifu and Kenshin, respectively, suggesting that they were associated with specific traits of the two genotypes, ncluding their HS responses. Several genes that were up-regulated in both inbred lines were confirmed by qRT-PCR (Fig 9). In contrast to the PI value changes observed in the microarray experiment, the induction revealed by qRT-PCR was less obvious, even though the expression levels clearly increased upon HS.

### Analysis of conserved *cis*-elements enriched for HSR genes

**Fig 10:**
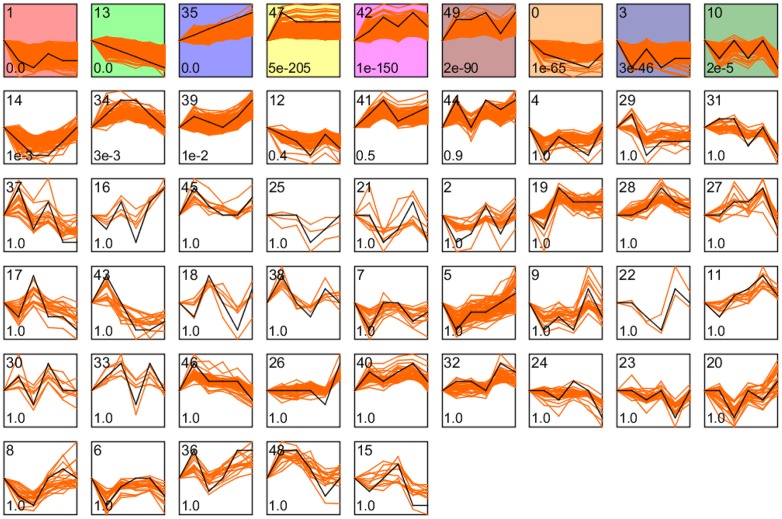
Short Time-series Expression Miner (STEM) clusters of expression profiles with HSR genes in Chiifu. The number of profiles in each cluster is at the top left corner of each STEM. Profiles are ordered based on the p-value significance of the number (at bottom-left corner) of assigned versus expected genes. Colored frame denotes significant profiles (P-value≤0.01). Each graph displays the mean expression pattern (black lines) of the profile genes.

Because the full genome sequence has already been reported for Chiifu (http://brassicadb.org/brad/), we performed an analysis of *cis*-elements of the HSR genes in order to extend our knowledge of HSR mechanisms in *B*. *rapa*. Although the genome sequence of Kenshin has not yet been published, results obtained using the Chiifu genome sequence should be sufficient to provide information about Kenshin because expression of most TFs exhibited similar patterns in both inbred lines (Fig 5). We performed our clustering analysis with STEM [37] using HSR genes that were > 2-fold DEGs (8,160 genes) in Chiifu. Fifty profiles were obtained from STEM, and nine of them significant overrepresentation (*p* < 0.01) (S23 Table, Fig 10). We then subjected the 1,000 bp upstream sequences of the genes in the nine significantly overrepresented profiles to PLACE database for motif scanning [38]. The occurrences of these motifs were compared with their frequencies among all promoters represented in the 135K microarray. A P-value was then calculated for each motif and profile combination, based on the hypergeometric distribution [39]. Motifs with P-values below 10^-4^ were considered to be significantly enriched significantly enriched motifs (SEMs) (S24 Table). Among the SEMs in profiles P35, P42, P47, and P19, we identified several motifs related to the HSR: CCAATBOX1 (CCAAT) in P35 is required for heat shock promoter activity [86,87]; and ACGTABREMOTIFA2OSEM (ACGTGKC) in P42, ABRELATERD1 (ACGTG) in P47, and two calcium-responsive motifs in P47, ABRERATCAL (MACGYGB) [88], and CGCGBOXAT (VCGCGB) could be responsible for the HS response. These three motifs are also known as ABREs binding by a bZIP TF responding to multiple stresses, such as drought and cold [88–93]. Our motif and transcriptome analyses suggest that ABREs are also necessary for the HSR. SEMs found in down-regulated genes were also related to the HSR: the DRECRTCOREAT (RCCGAC) motif, represented in P1 and P3, is an AP2 TF-binding site involved in cold and ABA-responsive processes [94]; and SITEIIATCYTC (TGGGCY), in P1 and P13, is a TCP-domain TF-binding site important for mitochondrial oxidative phosphorylation. Furthermore, we subjected genes containing the same *cis*-elements to agriGO for GO enrichment analysis. Processes including response to heat, heat acclimation, MAPKKK cascade, calmodulin binding, and regulation of oxygen and ROS metabolic process were represented by up-regulated genes containing shared *cis*-elements (S24 Table). In addition, shoot system development, respiratory burst during defense response, and very-long-chain fatty acid metabolic processes were represented by down-regulated genes containing shared *cis*-elements (S24 Table). All of this information will be useful because combinatorial analysis of promoters, co-expression networks, and transcriptome can provide further insight into gene functions and signaling pathways [39,95].

## Conclusion

Genome-wide analysis of the transcriptome following heat treatment in two inbred lines of Chinese cabbage provided important information regarding the HSR. **First**, even though Kenshin was more resistant to membrane leakage than Chiifu, the number of DEGs was higher in Chiifu. **Second**, expression of small Hsps was highly induced by HS treatment, whereas high-molecular weight Hsps exhibited constitutively high expression. **Third**, although expression of several upstream genes of the HSR was induced by HS treatment, most genes associated with the signaling pathway were constitutively expressed, suggesting that they are regulated at the protein level. **Fourth**, besides well-known TFs, many TFs and orphan genes seemed to be related to HSR in *B*. *rapa*. **Fifth**, most of the *B*. *rapa* HSR is likely to use mechanisms identified in other plants, but the critical or core genes could be different. To strengthen HSR mechanism in Chinese cabbage, it will be essential to perform combinatorial analyses of transcriptomes, proteomes, metabolomes, and small RNAs.

## Supporting Information

S1 Fig Expression of DEGs following HS treatment in either or both inbred lines. (TIFF)

S2 Fig RT-PCR analysis of HSFs and HSPs following HS treatment in both inbred lines, Chiifu and Kenshin. (TIFF)

S1 Table Primers used in the RT-PCR experiment. (XLSX)

S2 Table Primer sequences used in the qRT-PCR experiment. (XLSX)

S3 Table Summary of microarray experiments. Leaf discs were prepared from 4-week-old plants at 22°C (0) and subjected to 45°C for the indicated time (0.5–4 h). Probe intensity (PI) value are the means of two independent experiments. NA, no *Arabidopsis c*ounterpart.
(XLSX)

S4 Table Summary of genes up-regulated over 2-fold by HS in Chiifu. Leaf discs were prepared from 4-week-old plants at 22°C and subjected to 45°C for the indicated time. Probe values are the means of two independent experiments.
(XLSX)

S5 Table Summary of genes up-regulated over 2-fold by HS in Kenshin. Leaf discs were prepared from 4 week old plants at 22°C and subjected to 45°C for the indicated time. Probe values are the means of two independent experiments.
(XLSX)

S6 Table Summary of genes down-regulated over 2-fold by HS in Chiifu. Leaf discs were prepared from 4 week old plants at 22°C and subjected to 45°C for the indicated time. Probe values are the means of two independent experiments.
(XLSX)

S7 Table Summary of genes down-regulated over 2-fold by HS in Kenshin. Leaf discs were prepared from 4 week old plants at 22°C and subjected to 45°C for the indicated time. Probe values are the means of two independent experiments.
(XLSX)

S8 Table List of GO terms significantly enriched among genes up-regulated by HS in Chiifu and Kenshin. Yellow shading indicates the FDR value below 0.05 (significantly represented items).
(XLSX)

S9 Table List of GO terms significantly enriched among genes down-regulated by HS in Chiifu and Kenshin. Yellow shade indicates the FDR value below 0.05 (significantly represented items).
(XLSX)

S10 Table KEGG pathway maps of HSR genes in Chiifu and Kenshin. Gene No. represents *Arabidopsis* counterparts involved in each pathways.
(XLSX)

S11 Table Intrinsic genes expressed in Chiifu or Kenshin under normal growth conditions. Expression levels of all genes in this list changed by over 4-fold in both genotypes
(XLSX)

S12 Table KEGG pathway maps of intrinsic genes. Gene No. represents *Arabidopsis* homologs involved in each pathway.
(DOCX)

S13 Table Genes induced/repressed by HS treatment. (XLSX)

S14 Table Summary of expression of Hsf and Hsp genes on the 135K microarray. The total number of genes was 244 (38 for Hsfs and 206 for Hsps).
(XLSX)

S15 Table Expression of *B*. *rapa* POD genes (170) upon HS (45°C). (XLSX)

S16 Table Genes for calmodulins and related proteins that respond to HS (45°C). (XLSX)

S17 Table Expression of *B*. *rapa* kinase genes (2,110) upon HS (45°C). (XLSX)

S18 Table Expression of *B*. *rapa* phosphatase genes (450) upon HS (45°C). (XLSX)

S19 Table Expression profile of transcription factor genes upon HS treatment. (XLSX)

S20 Table Expression of marker genes for thermotolerance in Chiifu and Kenshin. (XLSX)

S21 Table Orphan genes in Br135K microarray (5,349 genes). (XLSX)

S22 Table Expression profiles of membrane leakage-related (MLR) genes. MLR genes were defined as those whose expression matters correlated negatively or positively with the membrane leakage pattern, i.e., genes should exhibit no significant change from no treatment to 1 h HS treatment, but should change after 2 h HS treatment (over 2-fold, up or down).
(XLSX)

S23 Table Short Time-series Expression Miner (STEM) clusters of expression profiles with heat-responsive genes in Chiifu. (XLSX)

S24 Table Significantly over-represented (p<10^-4^) cis-elements/motifs in DEGs by HS Chiifu. GO enrichment was carried out using agriGO. Significantly enriched GO items were represented (FDR≤0.05).
(XLSX)
