# Supplementary figures and images for: Global Gene-Expression Analysis to Identify Differentially Expressed Genes Critical for the Heat Stress Response in Brassica rapa
Source: PLoS One. 2015 Jun 23;10(6):e0130451. doi: 10.1371/journal.pone.0130451 (PMC4477974; doi:10.1371/journal.pone.0130451)

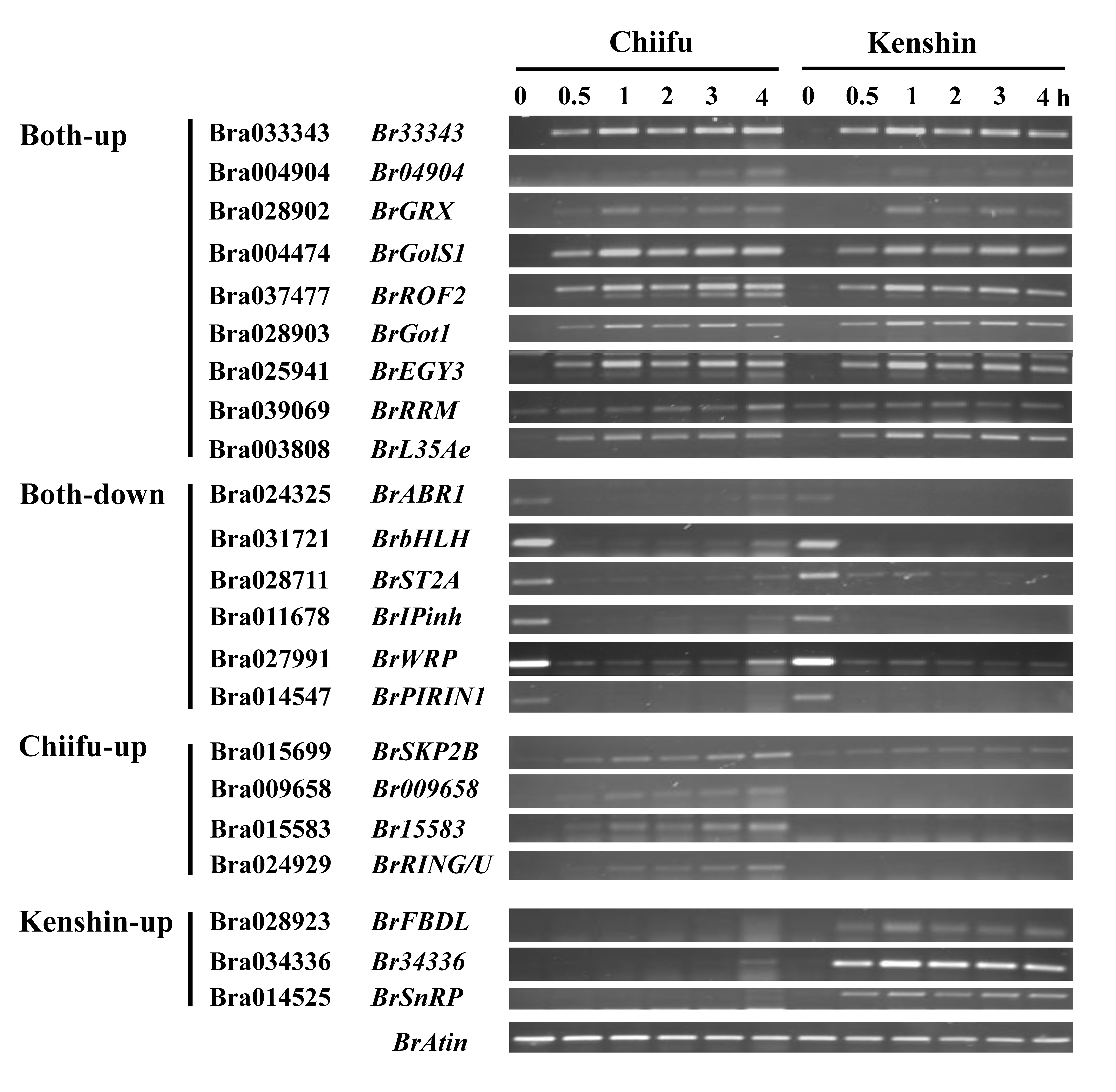

Supplement: S1 Fig — (TIFF) [file pone.0130451.s001.tiff]

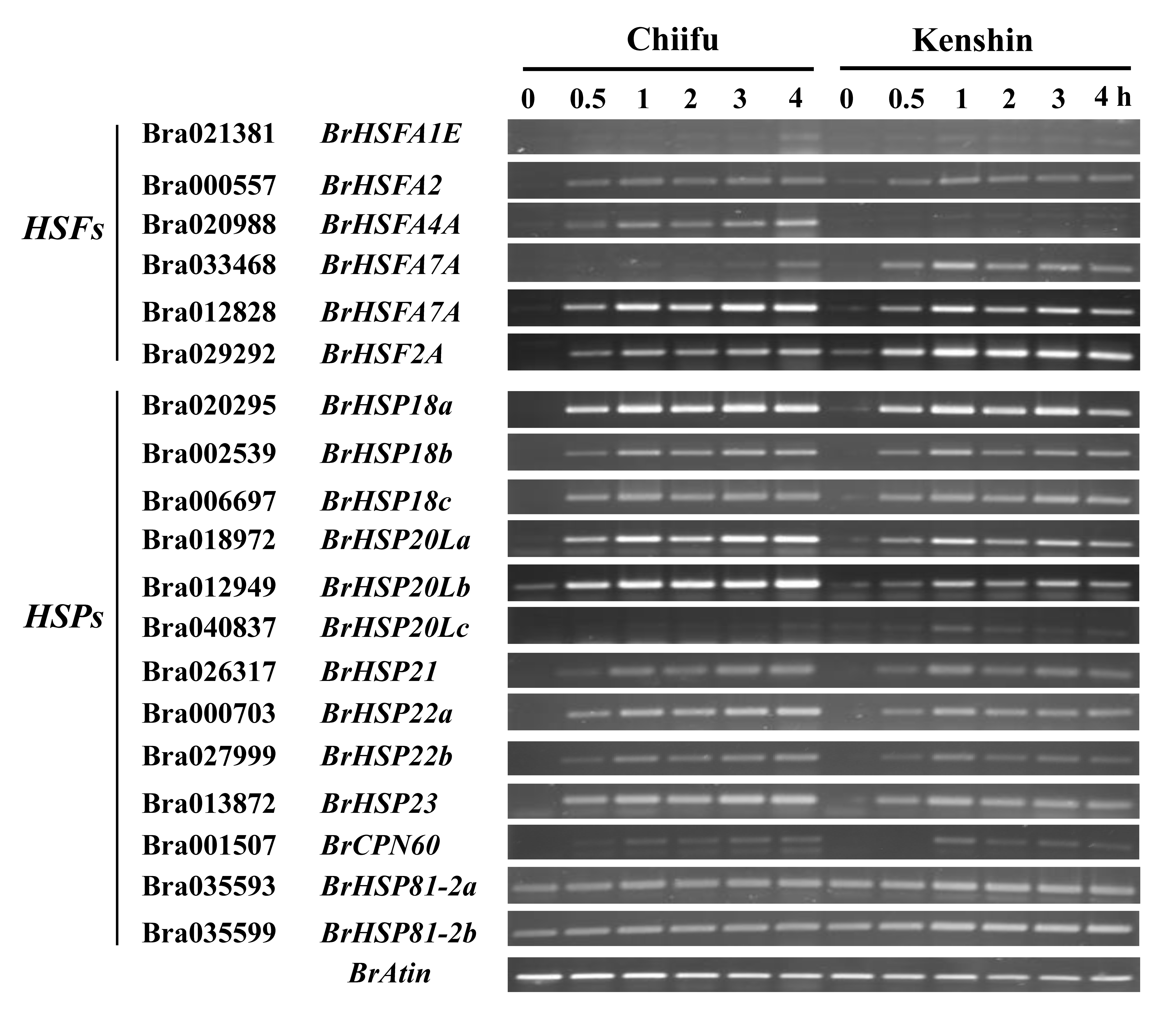

Supplement: S2 Fig — (TIFF) [file pone.0130451.s002.tiff]
