# Supplementary material for: Global Gene-Expression Analysis to Identify Differentially Expressed Genes Critical for the Heat Stress Response in Brassica rapa
Source: PLoS One. 2015 Jun 23;10(6):e0130451. doi: 10.1371/journal.pone.0130451 (PMC4477974; doi:10.1371/journal.pone.0130451)
Supplement: S12 Table — Gene No. represents Arabidopsis homologs involved in each pathway. (DOCX) [file pone.0130451.s014.docx]

**S12 Table**. KEGG pathway maps of intrinsic genes. The gene No. were represented by *Arabidopsis* homologs involved in each pathways.

| **KEGG_ID** | **Description** | **Gene No.** | |
| --- | --- | --- | --- |
|  |  | **Kenshin-up** | **Chiifu-up** |
| ath01100 | Metabolic pathways | 75 | 106 |
| ath01110 | Biosynthesis of secondary metabolites | 46 | 66 |
| ath01230 | Biosynthesis of amino acids | 20 | 18 |
| ath03010 | Ribosome | 8 | 18 |
| ath04075 | Plant hormone signal transduction | 13 | 17 |
| ath01200 | Carbon metabolism | 14 | 16 |
| ath04626 | Plant-pathogen interaction | 6 | 16 |
| ath00500 | Starch and sucrose metabolism | 6 | 14 |
| ath00480 | Glutathione metabolism | 4 | 10 |
| ath00940 | Phenylpropanoid biosynthesis | 2 | 10 |
| ath03018 | RNA degradation | 4 | 9 |
| ath00230 | Purine metabolism | 5 | 8 |
| ath00564 | Glycerophospholipid metabolism | 4 | 8 |
| ath03013 | RNA transport | 3 | 8 |
| ath00010 | Glycolysis / Gluconeogenesis | 10 | 7 |
| ath00520 | Amino sugar and nucleotide sugar metabolism | 7 | 7 |
| ath03040 | Spliceosome | 5 | 7 |
| ath04141 | Protein processing in endoplasmic reticulum | 7 | 7 |
| ath04145 | Phagosome | 5 | 7 |
| ath00240 | Pyrimidine metabolism | 4 | 6 |
| ath00260 | Glycine, serine and threonine metabolism | 3 | 6 |
| ath00270 | Cysteine and methionine metabolism | 3 | 6 |
| ath00280 | Valine, leucine and isoleucine degradation | 3 | 6 |
| ath00360 | Phenylalanine metabolism | 3 | 6 |
| ath00460 | Cyanoamino acid metabolism | 2 | 6 |
| ath00130 | Ubiquinone and other terpenoid-quinone biosynthesis |  | 5 |
| ath00400 | Phenylalanine, tyrosine and tryptophan biosynthesis | 3 | 5 |
| ath00410 | beta-Alanine metabolism | 3 | 5 |
| ath00903 | Limonene and pinene degradation | 3 | 5 |
| ath03015 | mRNA surveillance pathway |  | 5 |
| ath04712 | Circadian rhythm - plant | 4 | 5 |
| ath00040 | Pentose and glucuronateinterconversions | 4 | 4 |
| ath00330 | Arginine and proline metabolism | 10 | 4 |
| ath00380 | Tryptophan metabolism | 3 | 4 |
| ath00561 | Glycerolipid metabolism | 3 | 4 |
| ath00620 | Pyruvate metabolism | 5 | 4 |
| ath00630 | Glyoxylate and dicarboxylate metabolism | 3 | 4 |
| ath00945 | Stilbenoid, diarylheptanoid and gingerol biosynthesis | 2 | 4 |
| ath01210 | 2-Oxocarboxylic acid metabolism | 6 | 4 |
| ath01212 | Fatty acid metabolism | 1 | 4 |
| ath03008 | Ribosome biogenesis in eukaryotes | 2 | 4 |
| ath03030 | DNA replication |  | 4 |
| ath03430 | Mismatch repair |  | 4 |
| ath04070 | Phosphatidylinositol signaling system | 1 | 4 |
| ath04120 | Ubiquitin mediated proteolysis | 3 | 4 |
| ath00051 | Fructose and mannose metabolism | 3 | 3 |
| ath00053 | Ascorbate and aldarate metabolism | 2 | 3 |
| ath00073 | Cutin, suberine and wax biosynthesis | 1 | 3 |
| ath00250 | Alanine, aspartate and glutamate metabolism | 6 | 3 |
| ath00290 | Valine, leucine and isoleucine biosynthesis |  | 3 |
| ath00340 | Histidine metabolism | 4 | 3 |
| ath00350 | Tyrosine metabolism | 3 | 3 |
| ath00670 | One carbon pool by folate | 1 | 3 |
| ath00710 | Carbon fixation in photosynthetic organisms | 8 | 3 |
| ath00770 | Pantothenate and CoA biosynthesis |  | 3 |
| ath00900 | Terpenoid backbone biosynthesis | 1 | 3 |
| ath00906 | Carotenoid biosynthesis | 2 | 3 |
| ath00909 | Sesquiterpenoid and triterpenoid biosynthesis | 1 | 3 |
| ath00920 | Sulfur metabolism | 4 | 3 |
| ath00970 | Aminoacyl-tRNA biosynthesis | 1 | 3 |
| ath01040 | Biosynthesis of unsaturated fatty acids | 1 | 3 |
| ath03420 | Nucleotide excision repair |  | 3 |
| ath04122 | Sulfur relay system |  | 3 |
| ath04144 | Endocytosis | 6 | 3 |
| ath04146 | Peroxisome | 5 | 3 |
| ath00020 | Citrate cycle (TCA cycle) | 2 | 2 |
| ath00030 | Pentose phosphate pathway | 2 | 2 |
| ath00052 | Galactose metabolism | 2 | 2 |
| ath00061 | Fatty acid biosynthesis |  | 2 |
| ath00071 | Fatty acid degradation | 4 | 2 |
| ath00100 | Steroid biosynthesis | 2 | 2 |
| ath00190 | Oxidative phosphorylation | 7 | 2 |
| ath00310 | Lysine degradation | 2 | 2 |
| ath00430 | Taurine and hypotaurine metabolism | 2 | 2 |
| ath00511 | Other glycan degradation |  | 2 |
| ath00562 | Inositol phosphate metabolism | 2 | 2 |
| ath00563 | Glycosylphosphatidylinositol(GPI)-anchor biosynthesis | 1 | 2 |
| ath00592 | alpha-Linolenic acid metabolism | 2 | 2 |
| ath00600 | Sphingolipid metabolism |  | 2 |
| ath00650 | Butanoate metabolism | 1 | 2 |
| ath00750 | Vitamin B6 metabolism |  | 2 |
| ath00910 | Nitrogen metabolism | 3 | 2 |
| ath00950 | Isoquinoline alkaloid biosynthesis | 2 | 2 |
| ath00966 | Glucosinolate biosynthesis | 1 | 2 |
| ath02010 | ABC transporters |  | 2 |
| ath03020 | RNA polymerase | 1 | 2 |
| ath03060 | Protein export | 5 | 2 |
| ath03440 | Homologous recombination | 1 | 2 |
| ath00062 | Fatty acid elongation |  | 1 |
| ath00232 | Caffeine metabolism |  | 1 |
| ath00300 | Lysine biosynthesis | 2 | 1 |
| ath00450 | Selenocompound metabolism | 2 | 1 |
| ath00510 | N-Glycan biosynthesis | 1 | 1 |
| ath00531 | Glycosaminoglycan degradation |  | 1 |
| ath00565 | Ether lipid metabolism | 2 | 1 |
| ath00590 | Arachidonic acid metabolism | 1 | 1 |
| ath00640 | Propanoate metabolism |  | 1 |
| ath00730 | Thiamine metabolism |  | 1 |
| ath00780 | Biotin metabolism |  | 1 |
| ath00790 | Folate biosynthesis |  | 1 |
| ath00860 | Porphyrin and chlorophyll metabolism |  | 1 |
| ath00902 | Monoterpenoid biosynthesis |  | 1 |
| ath00904 | Diterpenoid biosynthesis |  | 1 |
| ath00908 | Zeatin biosynthesis | 1 | 1 |
| ath00941 | Flavonoid biosynthesis | 1 | 1 |
| ath00960 | Tropane, piperidine and pyridine alkaloid biosynthesis | 3 | 1 |
| ath01220 | Degradation of aromatic compounds | 1 | 1 |
| ath03050 | Proteasome | 2 | 1 |
| ath03410 | Base excision repair | 1 | 1 |
| ath03450 | Non-homologous end-joining |  | 1 |
